# Partnership With Families During Hospitalization of Persons With Dementia: Intervention and Measurement Strategies

**DOI:** 10.1093/geroni/igab046.1446

**Published:** 2021-12-17

**Authors:** Marie Boltz, Barbara Resnick, Judith Tate

## Abstract

Persons with dementia have high rates of hospitalization and are at risk for complications including psychological distress, and functional and cognitive decline. In turn, their family caregivers often face increased stress related to lack of preparedness to meet the complex needs of the patient during hospitalization and in the post-acute period. Hospitalization provides an opportunity to reframe the role of family caregivers from the traditional passive one to that of partners with the hospital team. The aim of the Family-centered, Function-focused Care (Fam-FFC) clinical trial is to test a nurse-family partnership model that incorporates a four step approach to optimize behavioral, functional, and cognitive outcomes in hospitalized persons with dementia and increase preparedness of caregivers to continue to optimize these outcomes in in the acute and post-acute recovery period. In this symposium we provide a description of the intervention with regard to theoretical support, four step process, and cultural appropriateness of the process. Two presentations describe, among Black and white dyads, evidence to support the psychometric properties of major outcome measures, caregiving preparedness and neuropsychiatric symptoms, in hospitalized dyads living with dementia. The final presentation describes a strategy to engage the dyad in goal development and evaluation, and its effect upon hospital readmissions. Findings from this symposium will help to identify intervention and measurement resources for those working with hospitalized persons with dementia and their family caregivers, and guide ongoing research needs in this area. Our discussant will synthesize the research findings and discuss implication for research, policy, and practice.

